# A Review: Does Complement or the Contact System Have a Role in Protection or Pathogenesis of COVID-19?

**DOI:** 10.1007/s41030-020-00118-5

**Published:** 2020-05-13

**Authors:** Natella Maglakelidze, Kristen M. Manto, Timothy J. Craig

**Affiliations:** 1 Penn State University College of Medicine, 500 University Drive, Hershey, PA 17033 USA; 2 Department of Medicine and Pediatrics, Penn State University, 500 University Drive, Hershey, PA 17033 USA

**Keywords:** Angiotensin receptors, Bradykinin, Coronavirus, COVID-19, Complement, Contact system

## Abstract

**Introduction:**

COVID-19 presentation may include a profound increase in cytokines and associated pneumonia, rapidly progressing to acute respiratory distress syndrome (ARDS). This so-called cytokine storm often leads to refractory edema, respiratory arrest, and death. At present, anti-IL-6, antiviral therapy, convalescent plasma, hydroxychloroquine, and azithromycin among others are being investigated as potential treatments for COVID-19. As the disease etiology and precise therapeutic interventions are still not definitively defined, we wanted to review the roles that complement and the contact system may have in either the treatment or pathogenesis of the disease.

**Methods:**

We searched the recent literature (PubMed) on complement and coronavirus; contact system and coronavirus; bradykinin and coronavirus; and angiotensin receptor and coronavirus. The manuscript complies with ethics guidelines and was deemed exempt from institutional review board approval according to Human Subjects Protection Office guidelines.

**Results:**

Mouse models are available for the study of coronavirus and complement. Although complement is effective in protecting against many viruses, it does not seem to be protective against coronavirus. C3 knockout mice infected with SARS-CoV had less lung disease than wild-type mice, suggesting that complement may play a role in coronavirus pathogenesis. Some evidence suggests that the observed pulmonary edema may be bradykinin-induced and could be the reason that corticosteroids, antihistamines, and other traditional interventions for edema are not effective. Angiotensin-converting enzyme 2 (ACE2) is a co-receptor for SARS-CoV-2, and studies thus far have not concluded a benefit or risk associated with the use of either ACE-inhibitors or angiotensin receptor antagonists.

**Summary:**

Activation of complement and the contact system, through generation of bradykinin, may play a role in the SARS-CoV-2-induced pulmonary edema, and our search suggests that further work is necessary to confirm our suspicions.

## Key Summary Points

**Table:** 

Is complement essential for protection against coronavirus? From experiments with C3 knockout mice, it does not seem to be important.
Is complement part of the pathogenesis of the infection? C3 knockout mice seem to suggest that the pathology is partially secondary to complement activation.
Does the contact system play a role in protection against the coronavirus? These data are not yet available.
Does the contact system play a role in the edema associated with pneumonia and ARDS in COVID-19? There are some preliminary data that suggest bradykinin-induced edema may be part of the pathogenesis of the viral infection.

## Effect of the Complement System on Coronavirus and COVID-19

**Fig. 1:**
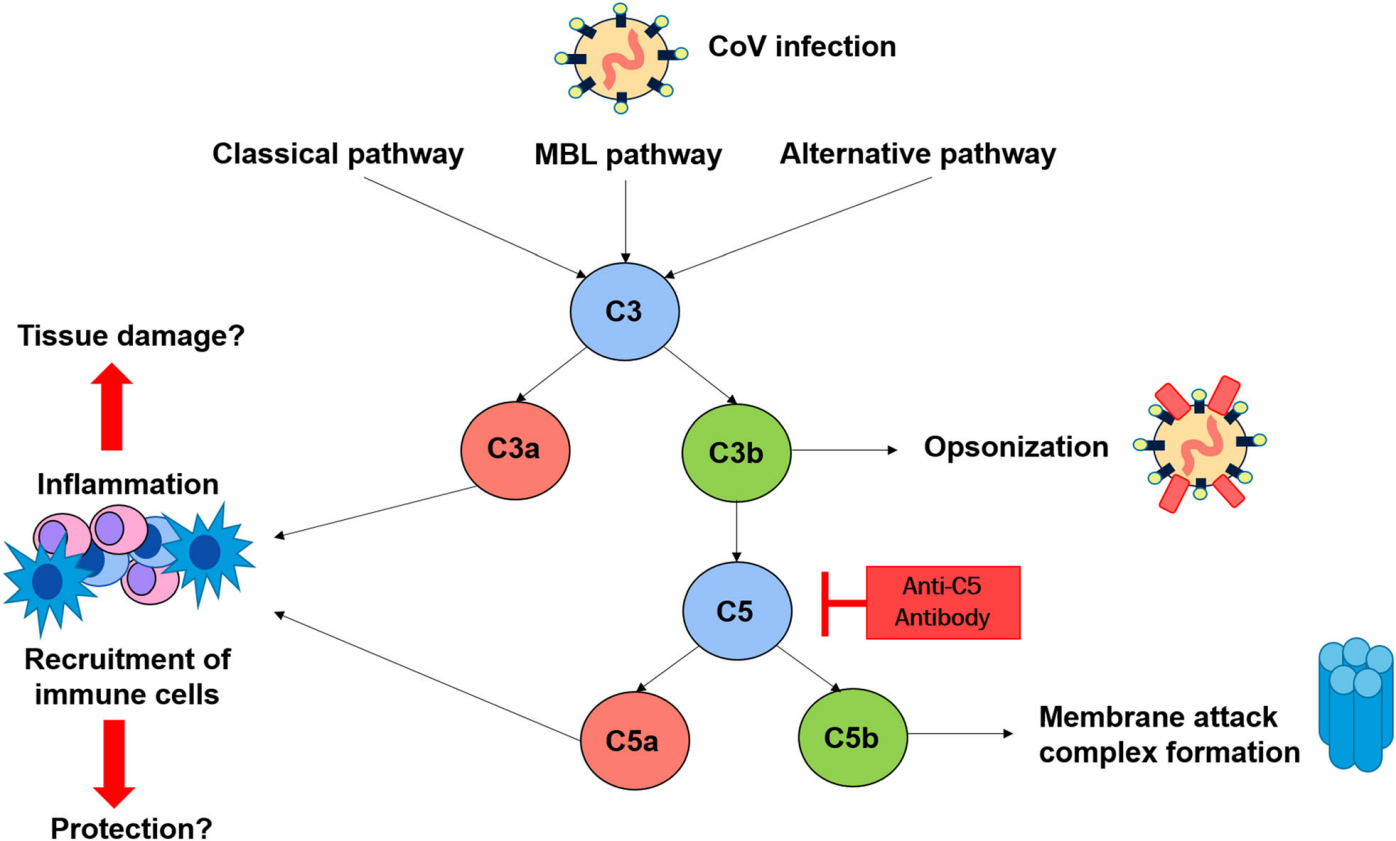
Effects of CoV-mediated complement activation and potential site of therapeutic inhibition. SARS-CoV and MERS-CoV have been shown to activate the complement pathway. Complement activation can occur through three distinct pathways (classical, MBL, and alternative) that converge at the C3 component. C3 can be converted into C3a and C3b. C3b mediates pathogen opsonization and activates the conversion of C5 into C5a and C5b. C5b mediates the formation of the membrane attack complex, which leads to cell lysis. C3a and C5a are known pro-inflammatory molecules that promote immune cell recruitment to the site of infection. Complement response to CoV is largely unclear, as it may be protective or pathogenic. Recent evidence suggests that eculizumab, a monoclonal antibody against C5, can be utilized to inhibit complement in response to CoV in order to reduce potential tissue damage and prevent disease exacerbation

The complement system plays a critical role in the rapid host innate immune response to bacterial, viral, and fungal infections [1]. Complement activation allows antibodies and phagocytic cells to detect and clear microbes at the site of infection and stimulates the recruitment of inflammatory cells, including macrophages, neutrophils, and mast cells [2, 3]. There are three distinct pathways through which the complement cascade can be triggered: the classical antigen–antibody complex pathway, alternative pathway, and mannose-binding lectin (MBL) pathway [1, 3]. These pathways can lead to microbe opsonization, inflammation, recruitment of immune cells, and cell lysis, and therefore must be tightly regulated given their potential to harm host tissues [3] (Fig. 1).

Coronaviruses (CoVs) are enveloped, positive-sense RNA viruses that can infect a wide range of hosts including pigs, camels, bats, cats, and humans [1]. According to the Centers for Disease Control and Prevention, seven CoVs are known to give rise to human disease. CoVs 229E, OC43, NL63, and HKU1 can cause mild upper respiratory tract symptoms, similar to the “common cold” [1, 4]. On the other hand, three CoVs recognized as SARS (severe acute respiratory syndrome)-CoV, MERS (Middle East respiratory syndrome)-CoV, and SARS-CoV-2 (COVID-19) have led to more severe clinical outcomes for infected patients [1] (Table 1). Typical symptoms induced by CoV infection include fever, headache, and cough [4]. Conversely, SARS-CoV, MERS-CoV, and COVID-19 may initially present asymptomatically, but can progress to pneumonia, shortness of breath, renal insufficiency and, in some cases, death [4]. When examining the histopathological changes that occur in pulmonary lesions of SARS-CoV patients, studies have described the presence of a nonspecific innate immune response and swelling [1, 4]. The severe inflammation that accompanies SARS-CoV infection can also lead to lung tissue necrosis and alveolar hyperplasia [1]. Such substantial damage to the lung epithelium suggests that the inflammatory response induced by SARS-CoV can significantly contribute to the course of the disease. Specifically, C3a and C5a, pro-inflammatory molecules generated by the complement system, can trigger inflammatory cell infiltration and contribute to lung injury [1]. Additionally, mounting evidence indicates that SARS-CoV infection can activate the complement cascade systemically, thus affecting other organs and influencing disease exacerbation [2].

**Table 1:** Pathogenicity and transmission of three coronavirus (CoV) outbreaks

Virus	Outbreak	Case fatality rate (%)	Pandemic	Status
SARS-CoV	2002–2004	9.5	Yes	Eradicated from intermediate animal reservoir
MERS-CoV	2012–	34.4	No	Continuous circulation in animal reservoir
SARS-CoV-2 (COVID-19)	2019–	3.4^a^	Yes	Efforts ongoing

SARS-CoV-2 (COVID-19) is the third coronavirus to emerge in the human population, following SARS-CoV and MERS-CoV. SARS-CoV-2 appears to be less pathogenic in comparison; however, its origin, transmission, and pathogenesis remain largely unknown. (Table newly created, but adapted from Munster et al. [16] and Rajgor et al. [17])

^a^Estimated by the World Health Organization as of March 3, 2020

Interestingly, the role of the complement system in SARS-CoV pathogenesis is considered controversial. In particular, multiple studies have investigated the importance of the MBL pathway of complement in the context of SARS-CoV infection; however, the results were contradictory and inconclusive. One study, published by Ip et al., demonstrated that patients with low serum MBL levels were at higher risk of becoming infected with SARS-CoV, suggesting that complement activation via MBL may be critical in protecting the host from SARS-CoV infection [5]. Conversely, Yuan et al. showed that there is no clear link between patients’ *MBL* genotypes/haplotypes and their susceptibility to SARS-CoV infection and disease [6]. Furthermore, some in vitro studies have revealed that MBL does not consistently bind to the SARS-CoV Spike protein, highlighting the uncertainty surrounding SARS-CoV recognition by complement [7]. Unfortunately, in vivo studies investigating the relationship between SARS-CoV pathogenesis and complement are lacking. While the scientific literature on MERS-CoV pathogenesis and complement response is not as extensive as that on SARS-CoV, studies have shown that inhibiting the complement system by blocking the C5a/C5a receptor can reduce MERS-CoV-mediated lung tissue damage in infected mice [8, 9]. Collectively, the discrepancies in the results of these studies reveal that complement response to CoV, SARS-CoV in particular, is largely unclear, as it may be protective or pathogenic [10].

Studies exploring the relationship between SARS-CoV infection and complement have demonstrated that complement activation can lead to disease exacerbation. Indeed, Gralinski et al. reported that intranasal infection of mice with mouse-adapted SARS-CoV resulted in activation of the complement cascade systemically and led to immune cell infiltration in the lung as early as 1 day post-infection [2]. In order to determine whether complement activation is involved in the pathogenic outcomes observed in patients infected with SARS-CoV, this study used mice genetically null for the gene *C3* (C3^−/−^) [2]. C3 is the major component of the complement system and is involved in all three complement pathways (Fig. 1). The study concluded that C3^−/−^ mice infected with SARS-CoV experienced less respiratory illness compared with infected wild-type mice [2]. Additionally, infected C3^−/−^ mice had decreased numbers of inflammatory neutrophils and monocytes, immune cells known to be implicated in CoV pathogenesis, recruited to their lungs. Finally, C3-deficient mice had lower serum and lung tissue cytokine levels than SARS-CoV-infected controls [2]. Collectively, these findings reveal that without complement, SARS-CoV is unable to induce as robust an inflammatory response as it does in wild-type mice. The exact mechanism through which SARS-CoV is recognized by complement is still under investigation; however, the results of this study suggest that SARS-CoV infection activates complement, which subsequently contributes to disease. Additionally, there is evidence that complement response to SARS-CoV also leads to potent inflammation systemically, as demonstrated by complement protein deposition in the kidneys [2]. Importantly, since the loss of C3 had no influence on the viral titer levels in mouse lung tissue, it suggests that the complement system may not be necessary for protection against SARS-CoV infection [2].

A recent manuscript by Campbell and Kahwash in *Circulation* called for initiating a trial of complement inhibition with the use of eculizumab, a monoclonal antibody against C5. The clinical observations of life-threatening COVID-19 include elevated lactate dehydrogenase (LDH), d-dimer, and bilirubin, decreased platelets, anemia, and renal and cardiac involvement, all of which are also seen in atypical hemolytic uremic syndrome (aHUS). Excessive complement activation leading to diffuse thrombotic microangiopathy (TMA) is the pathogenesis of aHUS. The end-organ dysfunction and the findings above, which respond to eculizumab, suggest that this intervention may also be successful in severe COVID-19 [11].

Overall, a better understanding of how complement interacts with SARS-CoV-2 and affects COVID-19 pathogenesis can lead to the development of more effective therapeutics for infected patients. If the complement system does in fact promote disease progression post-CoV infection, then inhibiting complement signaling may be an effective approach. In fact, antibodies against C5/C5a can potentially help reduce the pulmonary dysfunction observed in COVID-19 patients (Fig. 1). Thus, further investigation is needed in order to determine the mechanisms that govern SARS-CoV-2 pathogenesis and whether the complement system is associated with disease exacerbation.

## The Contact System and Coronavirus Infections

The contact system is regarded as a branch of the innate immune defense against microorganisms. There is an increasing list of pathogens that interact with contact factors, such as bacteria as well as fungi and viruses. In this way, it is a topic of interest when discussing coronavirus infections, especially in light of the recent COVID-19 pandemic and the increasing need for therapies and a vaccine. Although the literature surrounding the contact system and coronavirus is limited, there has been recent work about the roles of kallikrein and angiotensin-converting enzyme (ACE). In a preliminary study by Milewska et al., researchers studied the role of kallikrein 13 (KLK13) in HCoV-HKU1, a coronavirus discovered in Hong Kong in 2004 [14]. Human airway epithelial (HAE) cell cultures were incubated with HCoV. Based on mRNA expression, human tissue kallikreins (KLK) KLK1, KLK5, KLK6, KLK9, KLK12 and KLK14 were significantly expressed in the infected cells. In particular, HCoV-HKU1 infection was dependent on KLK13 activity, and HCoV-HKU1 did not replicate in HAE cells deficient in KLK13 [14]. Perhaps KLK13 inhibitors could serve as a novel therapy to prevent or treat coronavirus infections.

There is also an important role for ACE in coronavirus infection. In a 2003 *Nature* article, researchers identified a metallopeptidase, angiotensin-converting enzyme 2 (ACE2), isolated from SARS coronavirus (SARS-CoV)-permissive Vero E6 cells that efficiently binds to the S1 domain. The ability of the SARS-CoV S protein to mediate cell–cell fusion was dependent on the presence of ACE2 on neighboring cells. ACE2-transfected cells efficiently replicated on Vero E6 cells, with a visible cytopathic effect. Moreover, anti-ACE1 antibody had no observable effect on virally induced cytopathicity, whereas anti-ACE2 antibody inhibited cytopathicity in a dose-dependent manner. This work suggests that ACE2 contributes substantially to the efficiency of SARS-CoV replication and molecules or peptides that block ACE2, and may be a promising treatment [13].

As noted earlier, SARS-CoV-2 enters the cell via ACE2. ACE2 is a cell membrane-bound molecule that inactivates des-Arg bradykinin, which is a potent ligand of the bradykinin receptor type 1 (B1). The B1 receptor on endothelial cells is up-regulated by inflammatory cytokines, which is unlike the B2 receptor that is constituently expressed and is the receptor for bradykinin. Without ACE2 to inactivate des-Arg bradykinin bound to the B1 receptor, the lung is subject to angioedema [14].

A recent manuscript by van de Veerdonk et al. notes that as long as the virus persists, there is dysfunction of ACE2 function, leading to the dysregulation of the kinin-kallikrein pathway and thus causing the angioedema [15]. They further propose that blocking the B1 and B2 receptors may be beneficial in reducing the pulmonary edema noted in COVID-19. Some evidence that the edema is bradykinin-generated includes resistance to corticosteroids, epinephrine, and antihistamines. Unfortunately, though B2 receptor antagonists (icatibant) exist, there are presently no B1 receptor antagonists. It would be anticipated that dual B1 and B2 receptor inhibition would be ideal; however, the possibility that inhibiting the kallikrein-kinin pathway could be of benefit needs to be excluded [14, 15].

**Fig. 2:**
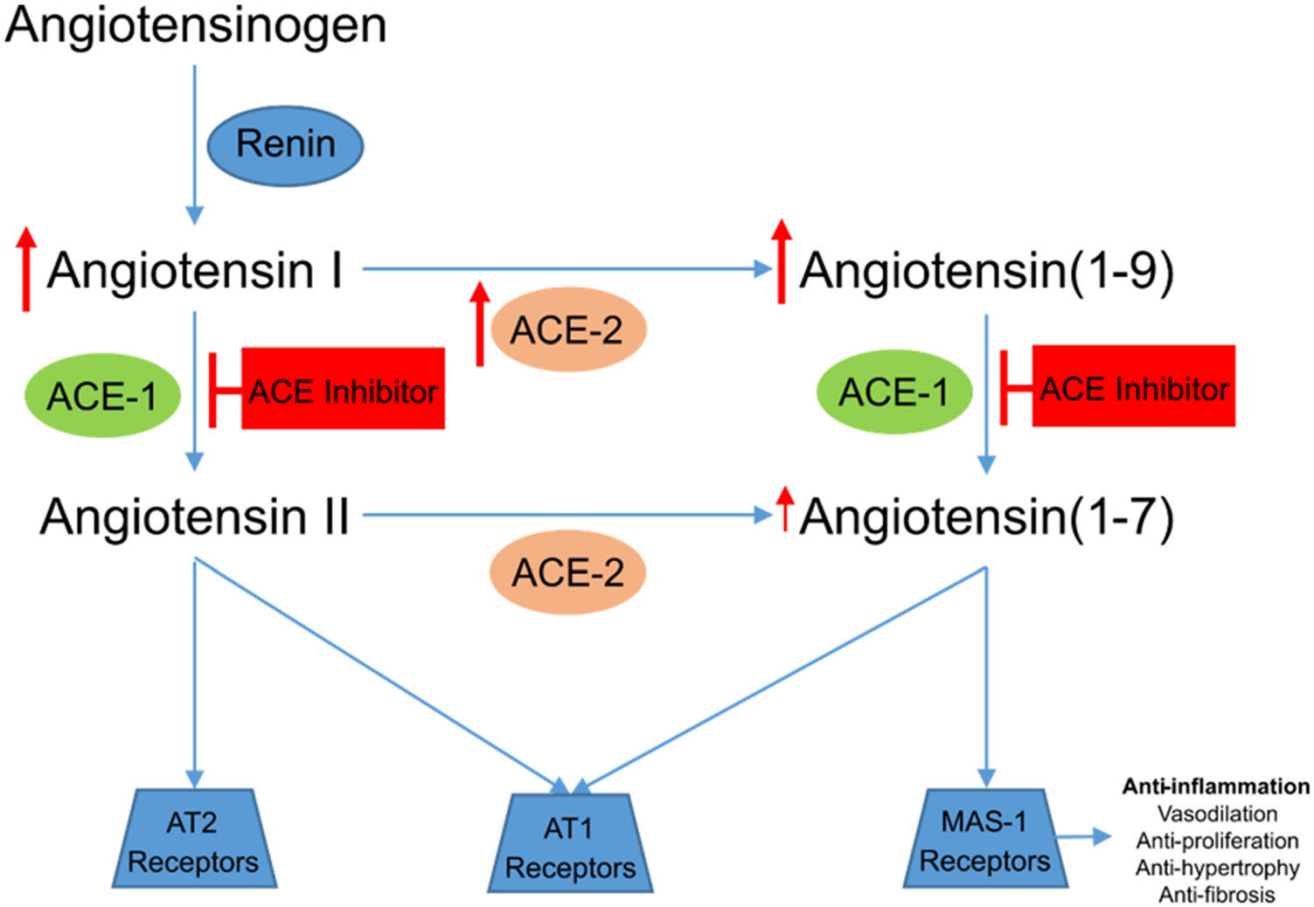
ACE1 inhibition in the setting of COVID-19 infection. ACE-inhibitors which target ACE1 lead to increased concentrations of angiotensin I. This may tend to up-regulate ACE2, which has been shown to modulate coronavirus entry and replication. As a result of increased ACE2, increased angiotensin (1-7) may produce anti-inflammatory effects via Mas receptors, which is not known to be beneficial in a viral infection

Given the body of literature surrounding the role of ACE in coronavirus infections, some have suggested using ACE1 inhibitors, such as enalapril and ramipril, and angiotensin receptor antagonists (ARBs), such as candesartan and valsartan, for COVID-19. However, despite three clinical trials being listed in progress in China, there is no trial evidence to date. Mechanistically, inhibition of ACE1 increases the concentration of circulating angiotensin I, which can be converted to angiotensin (Fig. 2). However, the conversion of angiotensin to angiotensin I is catalyzed by ACE1 and would not proceed to a large extent in the presence of an ACE inhibitor [12-15]. In fact, increased angiotensin I might tend to up-regulate ACE2, which has been shown in animal models. In addition, increased production of angiotensin from angiotensin II under the action of ACE2 could lead to increased anti-inflammatory activity, mainly through Mas receptor binding (Fig. 2), which some argue is harmful in a viral infection [14]. Ultimately, there needs to be more human data before we recommend these medications to patients for either the prevention or treatment of COVID-19.

## Summary

Both the complement and contact pathways seem to be potential targets for treating COVID-19. The only approved medication for humans that inhibits the complement cascade is eculizumab, which inhibits C5. At present, the best information that complement may contribute to disease pathogenesis comes from a C3-deficient mouse model. What we do not know is whether blocking C5, which follows C3, would be as effective in controlling the inflammation induced by the complement system. Evidence also suggests that inhibition of the contact system may be a worthy target, since there is at least some suggestion that production of bradykinin may add to the pulmonary edema seen in COVID-19. As for the decision to avoid or use ACE1s and ARBs, the data are not available to make a sound evidence-based decision, and thus continuing therapy as initially prescribed seems to be the only logical decision.

## References

[1] Li G (2020). Coronavirus infections and immune responses. J Med Virol.

[2] Gralinski LE (2018). Complement activation contributes to severe acute respiratory syndrome coronavirus pathogenesis. mBio.

[3] Janeway CA, Travers P, Walport M, Shlomchik MJ (2001). Immunobiology: the immune system in health and disease.

[4] Human Coronavirus Types. Centers for disease control and prevention. 2020. https://www.cdc.gov/coronavirus/types.html.

[5] Ip WK (2005). Mannose-binding lectin in severe acute respiratory syndrome coronavirus infection. J Infect Dis.

[6] Yuan FF (2005). Influence of FcgammaRIIA and MBL polymorphisms on severe acute respiratory syndrome. Tissue Antigens.

[7] Leth-Larsen R, Zhong F, Chow VT, Holmskov U, Lu J (2007). The SARS coronavirus spike glycoprotein is selectively recognized by lung surfactant protein D and activates macrophages. Immunobiology.

[8] Jiang Y (2018). Blockade of the C5a–C5aR axis alleviates lung damage in hDPP4-transgenic mice infected with MERS-CoV. Emerg Microbes Infect.

[9] Jiang Y (2019). Complement receptor C5aR1 inhibition reduces pyroptosis in hDPP4-transgenic mice infected with MERS-CoV. Viruses..

[10] Stoermer KA, Morrison TE (2011). Complement and viral pathogenesis. Virology.

[11] Campbell CM, Kahwash R (2020). Will complement inhibition be the new target in treating COVID-19 related systemic thrombosis?. Circulation.

[12] Aronson JK. Angiotensin converting enzyme (ACE) inhibitors and angiotensin receptor blockers in COVID-19. CEBM. 2020. www.cebm.net/covid-19/angiotensin-converting-enzyme-ace-inhibitors-and-angiotensin-receptor-blockers-in-covid-19/.

[13] Li W (2003). Angiotensin-converting enzyme 2 is a functional receptor for the SARS coronavirus. Nature.

[14] Milewska A (2020). Kallikrein 13: a new player in coronaviral infections. bioRxiv.

[15] van de Veerdonk F, et al. Kinins and cytokines in COVID-19: a comprehensive pathophysiological approach. 2020. 10.20944/preprints202004.0023.v1.

[16] Munster VJ (2020). A novel coronavirus emerging in China—key questions for impact assessment. N Engl J Med.

[17] Rajgor DD (2020). The many estimates of the COVID-19 case fatality rate. Lancet Infect Dis..

